# Effects of substrate annealing on the gold-catalyzed growth of ZnO nanostructures

**DOI:** 10.1186/1556-276X-6-566

**Published:** 2011-10-26

**Authors:** Christian C Weigand, Daniel Skåre, Cecile Ladam, Jostein Grepstad, Helge Weman

**Affiliations:** 1 Department of Electronics and Telecommunications, Norwegian University of Science and Technology, 7491 Trondheim, Norway; 2 Department of Physics, Norwegian University of Science and Technology, 7491 Trondheim, Norway; 3 SINTEF Materials and Chemistry, 7465 Trondheim, Norway

**Keywords:** zinc oxide, laser ablation, atomic force microscopy, thermal annealing, vapourliquid-solid growth, nanostructures; surface roughness, surface defects

## Abstract

The effects of thermal substrate pretreatment on the growth of Au-catalyzed ZnO nanostructures by pulsed laser deposition are investigated. C-plane sapphire substrates are annealed *prior *to deposition of a thin Au layer. Subsequent ZnO growths on substrates annealed above 1,200°C resulted in a high density of nanosheets and nanowires, whereas lower temperatures led to low nanostructure densities. Separate Au film annealing experiments at 700°C showed little variation in the size and density of the Au catalyst droplets with substrate annealing temperature. The observed variation in the density of nanostructures is attributed to the number of surface nucleation sites on the substrate, leading to a competition between nucleation promoted by the Au catalyst and surface nucleation sites on the rougher surfaces annealed below 1,200°C.

## Introduction

Semiconductor nanostructures have attracted great interest in the past decade due to a wide range of potential applications, e.g., in solar cells, lasers and sensors, and as building blocks of integrated systems [1-3]. Fabrication of different types of nanostructures, such as nanowires, nanorods, nanobelts and nanosheets can be achieved from a variety of methods, including solution-based, chemical and physical vapor deposition techniques, with and without the use of a metal catalyst [1,4-7]. The morphology and orientation of nanostructures can be controlled by tuning growth parameters such as the substrate temperature, background pressure and precursor flux, as well as the substrate material. Furthermore, substrate treatments like chemical etching and thermal annealing have been shown to have significant impact on nanostructure growth for solution-based and catalyst-free vapor deposition techniques [8-11]. For catalyst-assisted nanostructure growth, however, little information exists on the effects of substrate pretreatment *prior *to catalyst metal deposition [12]. Moreover, reports on pretreatment by thermal annealing often refer to substrates coated with a thin layer of the catalyst metal, resulting in alloying and formation of catalyst droplets, which serve to guide the subsequent nanostructure growth [13-16]. Thermal annealing of clean substrates, however, is often reported to cause smooth and well-defined step-and-terrace substrate surfaces [17,18].

In this report, we show how thermal annealing of the substrate *prior *to catalyst metalization can significantly impact catalyst-assisted nanostructure growth. This is demonstrated for ZnO nanostructures grown on c-plane sapphire substrates by pulsed laser deposition (PLD) using gold as a catalyst.

## Experimental work

The nanostructures were grown by ablation from a raster-scanned ZnO target using a 248-nm KrF excimer laser at 10 Hz repetition rate and a fluency of ~1.33 J/cm^2^. The substrates were heated to 700°C in a 0.5 mbar ambient of 5% oxygen/95% argon and ZnO was deposited for 30 min. Prior to growth, the "epi-ready" c-plane sapphire substrates (Valley Design Corp., 0° ± 0.25° miscut) were annealed in oxygen at 1,000, 1,200 and 1,400°C for 1 h, followed by deposition of a 1-nm-thin layer of Au using e-beam evaporation. No further annealing of the Au layer took place before introduction into the PLD chamber.

In order to investigate the effects of substrate annealing on the formation of Au catalyst droplets, separate experiments were carried out in the PLD chamber with a Au layer only. C-plane sapphire substrates coated with a 1-nm-thin film of Au were annealed for 5 min at the same growth temperature and ambient as for the growth of ZnO nanostructures.

The clean and Au-coated substrates were examined using atomic force microscopy (AFM), and the PLD-grown ZnO nanostructures were studied with scanning electron microscopy (SEM).

The size and density of the catalyst droplets resulting from the Au layer annealing experiments were determined using the image processing software "ImageJ" [19]. The analysis procedure adopting image contrast enhancement, noise removal and particle separation by threshold and "watershed" methods was applied to 2μm × 2μm AFM images of the Au droplets. From subsequent automated particle measurements, the area, circularity, diameter and number density of the Au droplets were calculated.

## Results and discussion

**Figure 1:**
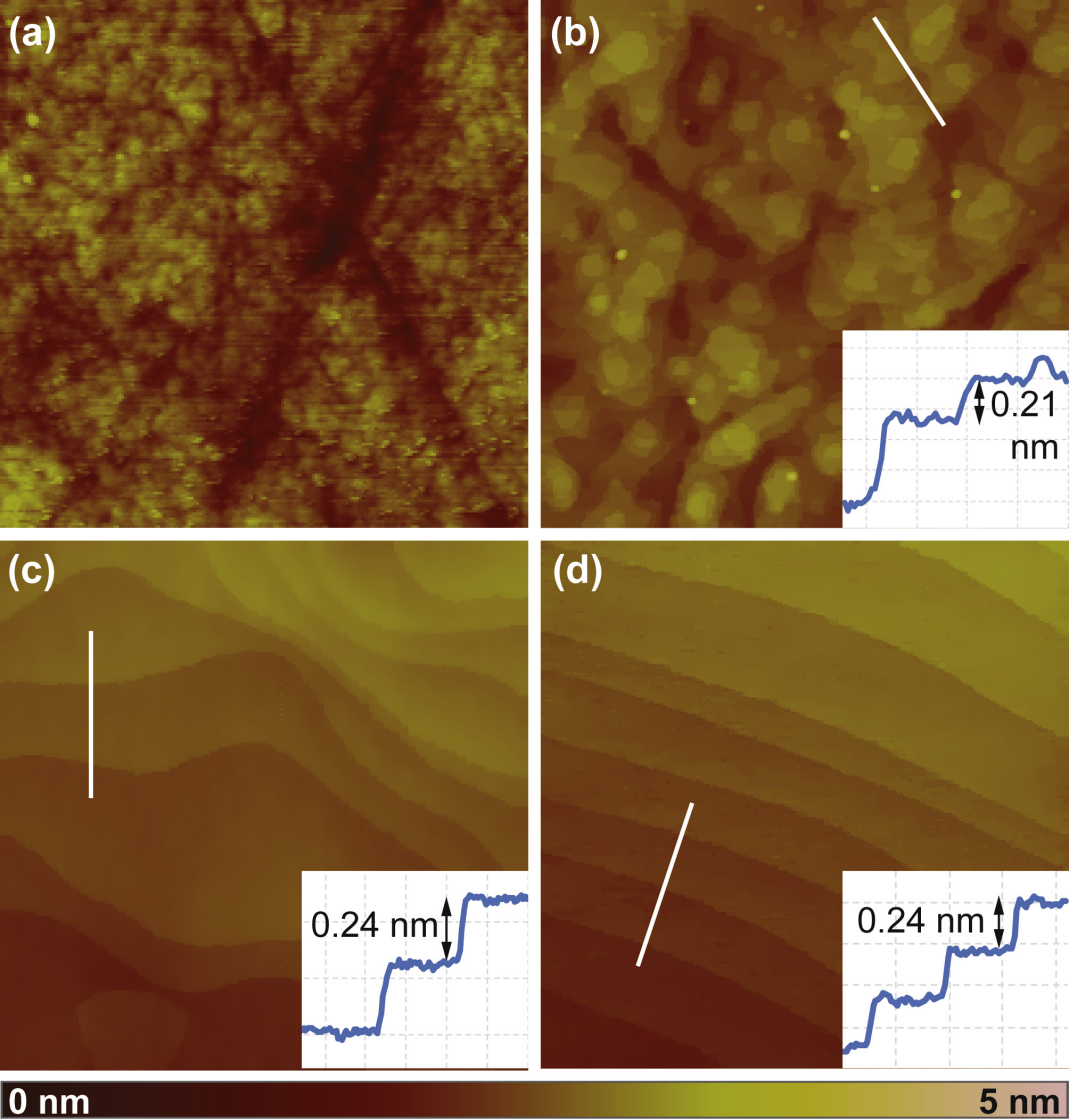
**Topography of c-plane sapphire substrates after thermal annealing**. 2μm × 2μm AFM images of c-plane sapphire substrate (**a**) as-received and after annealing at (**b**) 1,000°C, (**c**) 1,200°C and (**d**) 1,400°C. Insets in (**b**-**d**) show height profiles of the white lines. All AFM images use the same height scale (bottom).

The topography of the as-received and annealed c-plane sapphire substrates and their characteristic parameters are shown in Figure 1 andTable 1 respectively. Both as-received substrates and those annealed at 1,000°C exhibit a rough surface morphology with scratches from the surface polishing provided by the manufacturer (Figure 1a, b). While the as-received substrates show no sign of a step-and-terrace structure, the onset of terrace formation is observed upon substrate annealing at 1,000°C with distinct steps of varying height (cf. inset in Figure 1b). By increasing the annealing temperature to 1,200°C (Figure 1c), the substrate surface becomes atomically flat, displaying an irregular step-and-terrace morphology with constant step heights of about 0.24 nm, corresponding to atomic bilayers [17]. After substrate annealing at 1,400°C, the step-and-terrace morphology shows a distinct anisotropy with terraces of comparable widths and nearly parallel edges (Figure 1d).

**Table 1:** Characteristic features of substrates annealed at different temperatures and nanostructures grown on these substrates

Substrate anneal T (°C)	RMS roughness (nm)	Terrace width (nm)	Gold droplet size (nm)	Gold droplet density (cm^-2^)	Nanostructure density (cm^-2^)
As-received	0.46	N/A	24.5 ± 10.8	7.3 · 10^10^	2.53 · 10^8^
1,000	0.36	30-150	22.3 ± 10.2	7.0 · 10^10^	4.21 · 10^8^
1,200	0.24	100-700	24.0 ± 9.3	6.8 · 10^10^	1.19 · 10^9^
1,400	0.16	100-350	19.4 ± 6.5	1.4 · 10^11^	9.87 · 10^8^

**Figure 2:**
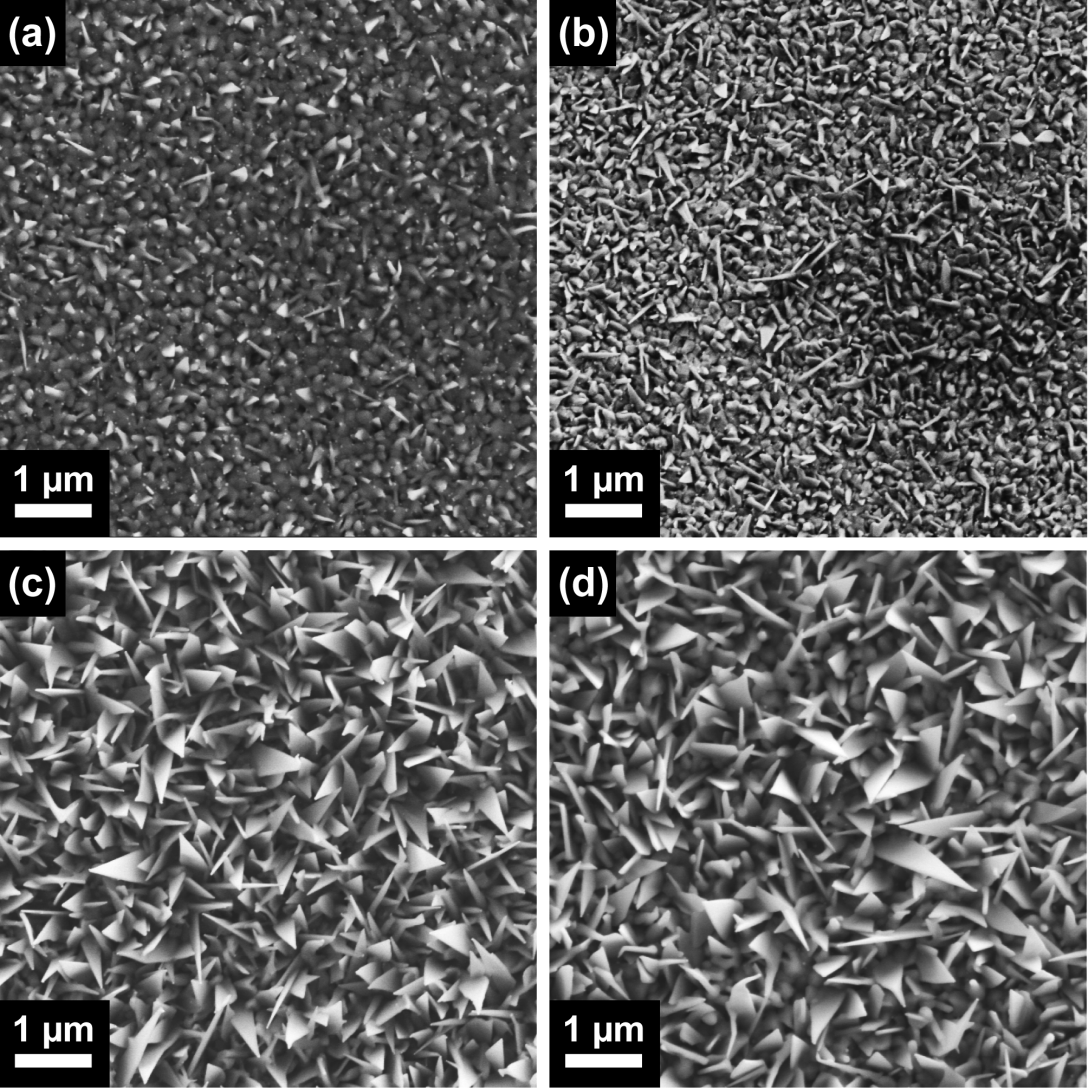
**ZnO nanostructures grown on annealed c-plane sapphire substrates**. Top-view SEM images of ZnO nanostructures grown on c-plane sapphire substrates (**a**) as-received and annealed at (**b**) 1,000°C, (**c**) 1,200°C and (**d**) 1,400°C.

Figure 2 shows SEM images of the ZnO nanostructures grown on sapphire substrates annealed at these different temperatures. Under the growth conditions adopted here, tilted ZnO nanowires and nanosheets form with the latter being the predominant type and Au particles could be clearly identified at the tip of these structures, indicating catalyst-assisted growth [20]. The size and density of the ZnO nanostructures grown on as-received sapphire substrates (Figure 2a) and on those annealed at 1,000°C (Figure 2b) were noticeably inferior to those grown on substrates annealed at 1,200°C and above (Figure 2c, d), i.e., on substrates with a step-and-terrace surface morphology (cf. Table 1). The latter exhibit a high density of nanosheets and nanowires with significantly increased sizes. We note that the annealing temperature seems to have no appreciable effect on the specific type of nanostructure grown. The observed differences in size and density could derive from two different parameters: (a) the size and density of the Au catalyst droplets and/or (b) the density of surface nucleation sites.

**Figure 3:**
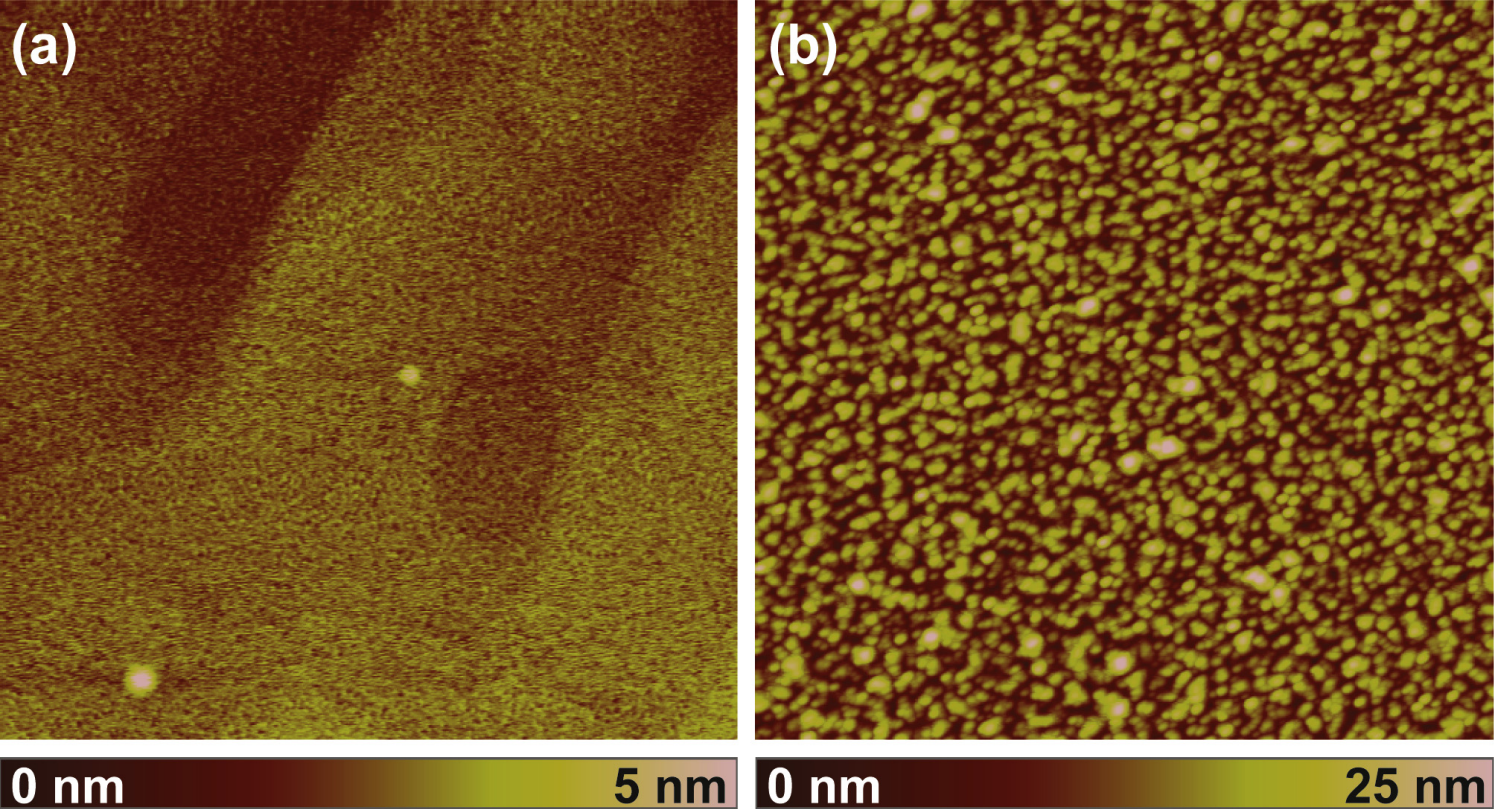
**Morphology of annealed c-plane sapphire substrates before and after annealing of the Au catalyst layer**. 2μm × 2μm AFM images of a 1-nm-thin Au layer on 1,200°C-annealed c-plane sapphire substrate before (**a**) and after annealing in the PLD chamber at 700°C for 5 min (**b**).

It has been reported that substrate pretreatment and morphology may significantly influence the size and density of Au catalyst droplets promoting nanostructure growth [12,21]. In order to investigate the effects of substrate annealing on catalyst droplet formation, Au-coated sapphire substrates were annealed in the PLD chamber at the 700°C growth temperature for 5 min, mimicking the adopted growth procedure prior to ZnO deposition. Before annealing of the Au layer, the step-and-terrace morphology of the substrate is still visible in AFM (Figure 3a). After annealing, the AFM images reveal a homogeneous distribution of Au droplets on the substrate surface regardless of the underlying step-and-terrace structure (Figure 3b). The size distribution and number density of the Au catalyst droplets are summarized in Table 1 and do not appear appreciably affected by thermal annealing of the substrate. Only for an annealing temperature of 1,400°C, we observe a slightly decreased average value and standard deviation of the droplet diameter as well as a higher number density of the Au droplets, presumably caused by the flat and smooth substrate topography [21]. We therefore conclude that the Au catalyst particle size is unlikely to bring about the observed differences in nanostructure size and density with substrate annealing temperature.

In the ideal scenario of catalyst-assisted nanostructure growth, the growth species are all incorporated into the nanostructure lattice via the catalyst-nanostructure interface. These species can reach the catalyst droplet either by direct impingement from the vapor or by surface diffusion from the substrate. In reality, however, the catalyst-nanostructure interface competes with nucleation at low-energy surface sites such as pits, craters and grain boundaries, as well as other surface defects abundant in rough surfaces [22-24]. At these surface sites, catalyst-free growth of ZnO is promoted, leading to reduced incorporation of growth species at the catalyst-nanostructure interface. Simultaneously, these surface sites also provide increased energy barriers for surface diffusion and thus imply reduced diffusion lengths of the adsorbed growth species [22].

**Figure 4:**
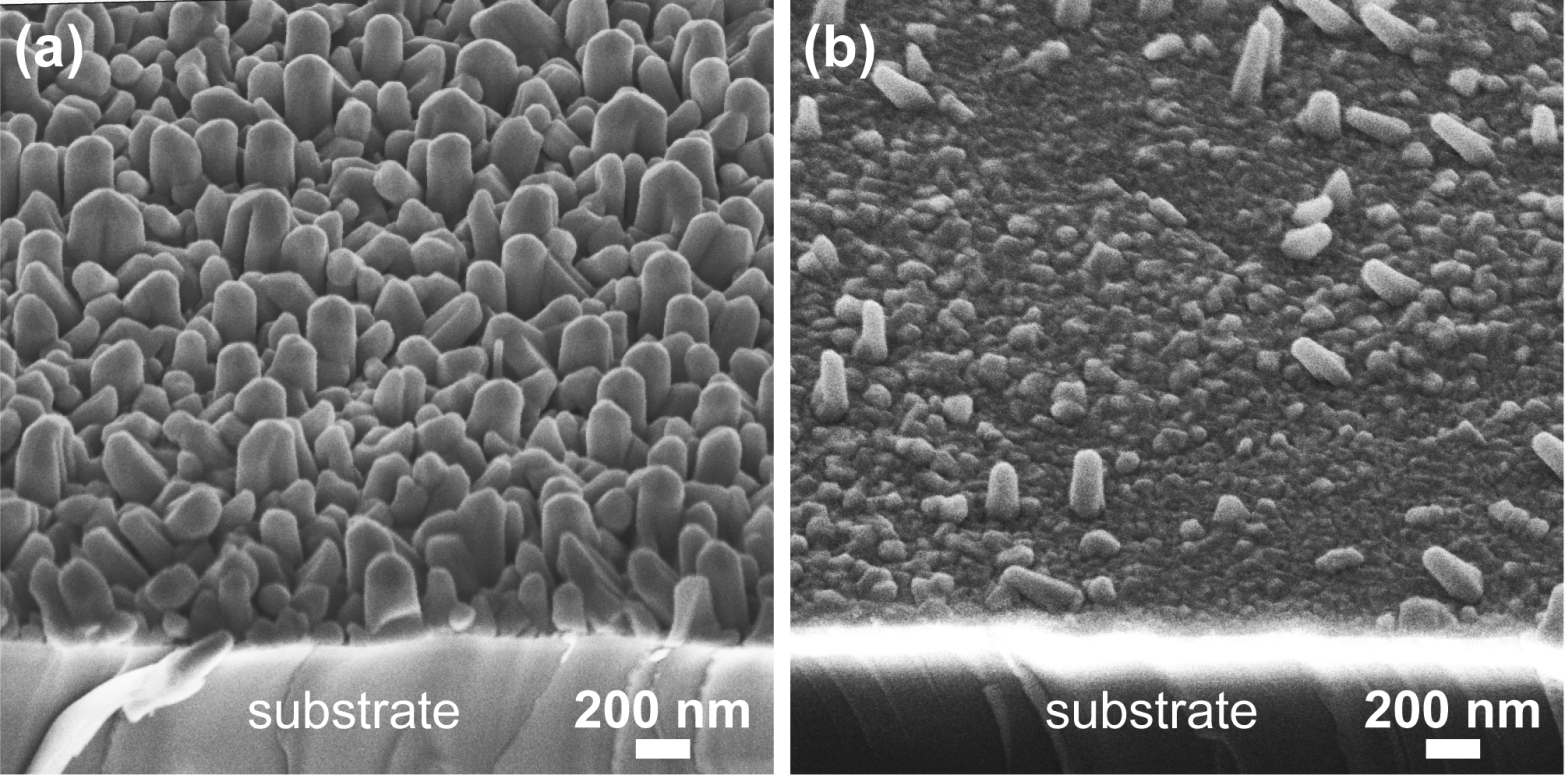
**ZnO grown on as-received and annealed substrates without the Au catalyst**. Tilted (45°) edge-on view SEM images of ZnO grown on c-plane sapphire substrates (**a**) as-received and annealed at (**b**) 1,200°C under identical growth conditions without the Au catalyst layer.

From Figure 1, it is apparent that the number of surface nucleation sites promoting catalyst-free growth is large for substrates annealed at 1,000°C and below. For the step-and-terrace structures formed at higher annealing temperatures, however, the density of surface nucleation sites is significantly reduced. This is also indicated by the decrease in the measured surface roughnesses with annealing temperature listed in Table 1. In order to investigate the effect of surface nucleation sites, we have also deposited ZnO without the metal catalyst layer, but otherwise identical growth conditions and procedures. The SEM images displayed in Figure 4a and 4b, respectively, show that a high density of catalyst-free ZnO nanorods grows on the as-received c-plane sapphire, whereas only few rods nucleate on the substrate annealed at 1,200°C. This implies a higher density of nucleation sites on as-received c-plane sapphire substrates compared to those annealed at 1,200°C, thus promoting enhanced catalyst-free growth of ZnO nanorods. For substrates annealed at 1,000°C and below, catalyst-assisted growth of ZnO nanostructures may therefore be obstructed by the increased competition for growth species with catalyst-free ZnO growth nucleated at these surface sites, leading to the observed low density and reduced size of ZnO nanostructures. Conversely, the small number of surface nucleation sites on the atomically flat surfaces of substrates annealed at 1,200°C and above allows for the growth species to reach the Au droplets without being incorporated at surface nucleation sites, thus promoting the increased density and larger sizes of ZnO nanostructures.

Furthermore, it is obvious from Figure 4 that the rate of catalyst-free ZnO growth is significantly higher on the as-received substrate than on sapphire annealed at 1,200°C. It has been previously reported that high rates of catalyst-free, vapor-solid (VS) growth can obstruct the catalyst-assisted growth of oxide nanostructures due to competition between the two growth modes [25]. This provides further support for the scenario proposed in the present work.

## Conclusion

In this study, we have shown that thermal annealing of the substrate prior to the Au catalyst deposition affects the density of ZnO nanostructures grown on c-plane sapphire. However, this substrate annealing does not seem to have a significant impact on the nanostructure morphology or the size and location of the Au catalyst droplets. The observed difference in nanostructure size and density can be explained by the competition between nucleation at the Au-ZnO interface and nucleation at low-energy surface sites associated with defects on rough substrate surfaces. The atomically flat surfaces obtained by high-temperature annealing promote formation of high densities of ZnO nanostructures through a significant reduction in surface nucleation sites, thus demonstrating the importance of smooth surfaces for catalyst-assisted nanostructure growth. We believe these findings will help improve control and understanding of catalyst-assisted nanostructure growth, also beyond the ZnO material system.

## Competing interests

The authors declare that they have no competing interests.

## Authors' contributions

CW participated in the acquisition of scanning electron microscopy images, in the analysis and in the interpretation of the data and drafted the manuscript. DS carried out the growth of the zinc oxide nanostructures and the acquisition of atomic force microscopic and scanning electron microscopic data. He also contributed to data analysis. CL conceived and designed the study and participated in the analysis and interpretation of the data. JG participated in the coordination of the study and helped to draft the manuscript. HW participated in conceiving, designing and coordinating the study and helped in the writing process of the manuscript.
